# Use of Reflectance Ratios as a Proxy for Coastal Water Constituent Monitoring in the Pearl River Estuary

**DOI:** 10.3390/s90100656

**Published:** 2009-01-23

**Authors:** Li-Gang Fang, Shui-Sen Chen, Dong Li, Hong-Li Li

**Affiliations:** 1 Department of Computer Engineering, Suzhou Vocational University, Suzhou 215104, P.R. China; E-Mails: flight1027@163.com (L.F.); lhl@jssvc.edu.cn (H.L.); 2 Public Laboratory of Environmental Science and Technology of Guangdong Province, Guangzhou Institute of Geography, Guangzhou 510070, P.R. China; 3 The Water Supply Corporation of Panyu District, Guangzhou 511400, P.R. China; E-mail: pywlab@vip.sina.com (D.L.)

**Keywords:** Pearl River Estuary, salinity, total suspended solid, colored dissolved organic matter, Hyperion imagery

## Abstract

Spectra, salinity, total suspended solids (TSS, in mg/L) and colored dissolved organic matter (CDOM, ag(400) at 400 nm) sampled in stations in 44 different locations on December 18, 19 and 21, in 2006 were measured and analyzed. The studied field covered a large variety of optically different waters, the absorption coefficient of CDOM ([ag(400)] in m^-1^) varied between 0.488 and 1.41 m^-1^, and the TSS concentrations (mg/L) varied between 7.0 and 241.1 mg/L. In order to detect salinity of the Pearl River Estuary, we analyzed the spectral properties of TSS and CDOM, and the relationships between field water reflectance spectra and water constituents' concentrations based on the synchronous in-situ and satellite hyper-spectral image analysis. A good correlation was discovered (the positive correlation by linear fit), between in-situ reflectance ratio R_680_/R_527_ and TSS concentrations (R^2^ = 0.65) for the salinity range of 1.74-22.12. However, the result also showed that the absorption coefficient of CDOM was not tightly correlated with reflectance. In addition, we also observed two significant relationships (R^2^ > 0.77), one between TSS concentrations and surface salinity and the other between the absorption coefficient of CDOM and surface salinity. Finally, we develop a novel method to understand surface salinity distribution of estuarine waters from the calibrated EO-1 Hyperion reflectance data in the Pearl River Estuary, i.e. channels with high salinity and shoals with low salinity. The EO-1 Hyperion derived surface salinity and TSS concentrations were validated using *in-situ* data that were collected on December 21, 2006, synchronous with EO-1 Hyperion satellite imagery acquisition. The results showed that the semi-empirical relationships are capable of predicting salinity from EO-1 Hyperion imagery in the Pearl River Estuary (RMSE < 2‰).

## 1. Introduction

The use of visible signatures in estuarine environments presents many difficulties due to the variety of optically active constituents present in estuarine waters. These constituents generally fall into three main classes: total suspended solids (TSS), colored dissolved organic matter (CDOM) and chlorophyll pigments (Chl_a). Each class is characterized by specific inherent optical properties (IOPs) relating to absorption and scattering processes [1]. CDOM is part of the dissolved organic matter, which can be classified into two major groups–natural organic matter and anthropogenic organic matter (AOM, i.e.; agricultural waste, and industrial organic pollutants). A fast development in industry and agriculture has led to large amounts of AOM being released into the water of Pearl River Estuary (PRE), which results in significant divergence in the composition and concentration of CDOM. These parameters (concentration and composition of CDOM) provide information on organic pollution and on their corresponding sources [2]. Solids suspended in the Delta River and estuaries are comprised of living and dead phytoplankton, mineral particles, and organic detritus. The particles have important optical effects on the upper layer of the river surface water. Suspended solid particles have different responses to salinity under different water quality conditions in the estuary. Each component is likely to change temporally and spatially due to flocculation and the breakup of suspended particles, which depends on a variety of parameters such as salinity, suspended sediment concentration, turbulence, organic matter content, and biochemical processes [3]. The approach we applied here, the quantitative derivation from hyperspectral data of useful geophysical parameters (mainly TSS, salinity) necessitates models of the IOPs and of the radioactive transfer [4, 5].

The Pearl River is the third largest river in China. The annual variation in its discharge depends significantly on the amount of rainfall it receives in the catchment, and from October to the next February the mean rainfall is comparatively low with about 30 mm/month, which results in the reduced runoff and causes marine water intrusion. During 2003–2006, the severe marine water intrusion events occurred around Macau, Zhongshan and Guangzhou area. The marine intrusion not only results in salinity increase in the rivers, influencing the freshwater taking sites along the river by delta cities, but also influences the concentration and distribution of nutrimental salt in the rivers. This leads to the change in the physical and chemical environment of the estuarine ecosystem. Consequently, the structure, function and health of the estuarine ecosystem could be affected as a result of such marine intrusion. Therefore, the effective approaches that can meet the needs of spatial and temporal coastal water monitoring are critical towards understanding the characteristics of marine intrusion into estuarine ecosystems. Although Brando and Dekker *et al.* had used Hyperion for water quality monitoring for quite a while, they made no reference to the analysis of salinity [6]. Miller and McKee established a robust linear relationship between band 1 (620–670 nm) of MODIS Terra 250 m data and *in situ* measurements of total suspended matter [7]. Hu *et al.* used MODIS for estuary water quality estimations and the research significantly improved our understanding of the global ocean. Moreover, they found the slope of the CDOM-salinity relationship changed with location (salinity range only: 24–32) within the bay. Besides, limitations become apparent when these data are applied to estuarine environments e.g.; the 1-km spatial resolution [8]. It is obviously worthwhile to develop new methodologies for effective marine intrusion monitoring in PRE, even though some literature already exists discussing the problem of the inversion of TSS, CDOM, and Chl_a based on in situ reflectance in the PRE [9]. Studies on TSS, CDOM and salinity by remote sensing in the PRE are limited, although several studies of optical and fluorescent characteristics of CDOM and salinity in Lingding Bay or coastal seawater have been reported [10, 11]. Moreover, the coarse resolution of Sea-viewing Wide Field-of-view Sensor (SeaWiFS) for detecting yellow substance according to existing research is not capable of monitoring the intrusions of marine waters [12]. So, there is a necessity in the research of optical characteristic and remote sensing of river intrusions in marine and coastal water, especially in salinity related river water color mechanisms. This is crucial for the understanding and monitoring of severe marine water intrusion in the PRE. Since 2006, the coastal agricultural managers in the PRE, such as Panyu District, have begun to adjust the local agricultural systems in order to reduce the influence of gradually severe marine intrusion on farming systems due to the longer and frequent marine intrusions [13].

## 2. Methods

### 2.1. Field Work

**Figure 1.**
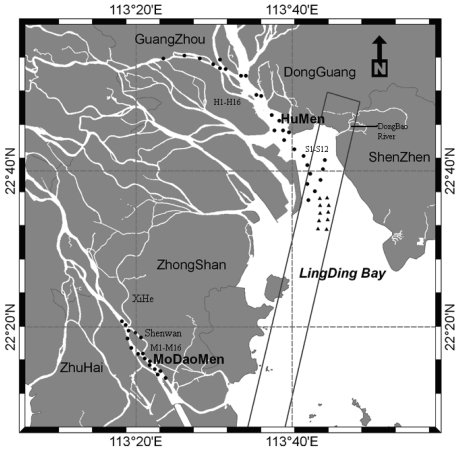
Map of water system in the Pearl River Estuary (PRE) and the black frame outlines map of EO-1 Hyperion footprint. Sample for spectra, salinity, TSS and CDOM in stations including 44 locations were measured and analyzed on December 18, 19 and 21, in 2006. There were 16 sampling locations in the Humen riverway (H1-H16), 16 locations in the Modaomen riverway (M1-M16) and 12 locations in the Lingding Bay (S1-S12). Additionally, triangles indicate the ten sampling sites synchronous with EO-1 Hyperion satellite imagery acquisition.

The field measurements of water surface spectra were carried out from 9:30 am to 2:30 pm (Beijing Time, GMT + 8) on December 18, 19 and 21, in 2006. Samples for spectra, salinity, TSS and CDOM in stations including 44 locations were measured and analyzed. Additionally, there were ten sites, synchronous with EO-1 Hyperion satellite imagery acquisition, where surface water was sampled and TSS concentrations and salinity were analyzed. The accurate positions of all the sampling stations were recorded by the global positioning system, as shown in Figure 1. We collected water samples during a cruise, while Panyu District Waterworks of Guangzhou and Zhongshan Water Conservancy Bureau collected water samples at locating monitoring sites during the fieldwork. All the samples were kept in an icebox and transported to a water quality analysis laboratory in Guangzhou as soon as possible in polypropylene sampling bottles. The entire procedure, including sample collection, storing, and measurement, was performed strictly according to the Technical Specifications Requirements for Monitoring of Surface Water and Waste Water of China (TSS, Chl_a; HJ/T 91—2002) [14], and Ocean Optics Protocols Version 2.0 (CDOM), distributed by NASA [15]. Absorption by CDOM was measured by first filtering samples with 25-mm Whatman GF/F glass-fiber filters and then measuring the absorption spectrum of the filtrate. Quartz cells with a path length of 10 cm were used in these measurements and distilled water was used as a reference. The absorption coefficient was calculated in the same manner. The absorption coefficient at 400 nm, ag(400) (m^-1^), is adopted in the study. TSS (mg/L) was determined by filtering samples onto 0.45 mm filters of known weight, drying them and measuring the weight gain due to sediment concentration. Surface salinity was measured using an YSI® 30/Set, a portable salinity temperature and conductivity meter. Elaborate precautions during analysis were taken to avoid contamination. In other words, each water constituent analysis was carried out in the Center of Water Environment Monitoring of Pearl River (Guangzhou) according to the criterion reported in other studies [10].

### 2.2. The Measurement and Processing of Field Spectra

#### 2.2.1. Spectral Measurement

Spectral measurements of radiance at the water's surface were carried out using ASD FieldSpec PR manufactured by the ASD Company in the USA. The instrument operates in the bands ranging from 350 to 2500 nm, with wavelengths resolution of 1 nm. To reduce the effect of water's mirror reflection, an observation angle method was adopted [16] and the boat's shadow was avoided by facing the boat side measured to Sun. In the spectral measurement, observation azimuth angle was less than 135° (sun azimuth angle is considered as 0°), and observation zenith angle was 45°. In each station, three important parameters were measured: radiance of water surface, sky and reference board. Integral time of the instrument was defined as 172 ms and 10 spectral curves were continuously measured at a time. Standard board with a reflectance of 30% was adopted and spectral measurements were taken from 0930 to 1430. The spectral measurements were always synchronous with water sample collection. This precaution can reduce errors in computing water leaving radiance values from measured radiance values due to variation in solar zenith angle.

#### 2.2.2. Spectral Processing

Radiance of surface water at each station was calculated by averaging the measured data (the abnormal values caused from the capillary waves at river surface were eliminated during data analysis). Generally, the surface reflectance of water is calculated by the ratio of reflection radiance and incidence radiance in water quality remote sensing monitoring. In fact, the surface reflectance of water contains not only the reflection information of water but also information of diffused sunlight reflected by air-water interface. Thus, to obtain pure reflectance information of water, we converted *in-situ* radiance of water to remote sensing reflectance by Equation (1), (2), and (3) that are described in detail below.

Water leaving radiance is spectral information of water reflected by air-water interface. Point-blank reflection of sunlight can be avoided with the observation azimuth of 135 degree and water radiance (L_sw_) measured by ASD can be calculated by Equation (1):

(1) $${\text{L}}_{\text{sw}}={\text{L}}_{\text{W}}+\text{r}\times L_{\text{sky}}$$

where *L_W_* is water leaving radiance, *L_sky_* is information of diffused sky light (involving no water information) and r is the sky light reflected by air-water interface. The value of r is usually between 2.1% and 5%, depending on the sun's position (θ_0_, Φ_0_), geometric conditions of observation (θ_v_, Φ_v_), wind speed, wind direction and roughness degree of water surface. Because average wind speed was less than 3 m/s and wind speed of most stations was less than 5 m/s during field measurements of water spectra, the value of r was defined as 0.022 [16].

Remote sensing reflectance was calculated by Equation (2):

(2) $$R_{rs}=L_{W}/E_{\text{d}}(0^{+})$$

where, *E*_d_ (0^+^) is the total incidence radiance of water surface it can be calculated by *L_p_* [Equation (3)]:

(3) $$E_{\text{d}}(0^{+})=\pi L_{p}/\rho_{p}$$

where, ρ*_p_* is reflectance of reference board, L_p_ is radiance of reference board.

### 2.3. Remote Sensing Data

#### 2.3.1. Imagery Acquisition

Hyperion imagery of Pearl River Estuary was acquired at 1037 (local standard time) on 21 December 2006, south China. The instrument is onboard the EO-1 satellite [17] that was launched on 21 November 2000, and its imagery data are recorded in 16-bit radiance values.

Hyperion is the first earth-orbiting imaging spectrometer operating across the full solar-reflected spectrum, collecting 196 unique calibrated spectral channels ranging with spectral coverage from 426-2395 nm and 10-nm sampling and spectral response functions. The spatial sampling is 30 m with a 7.7-km imagery swath and 185-km length, which is well suited for detecting and portraying complex spatial distributions of salinity and other water constituents in salt water intrusion region.

#### 2.3.2. Imagery Pre-Processing

**Figure 2.**
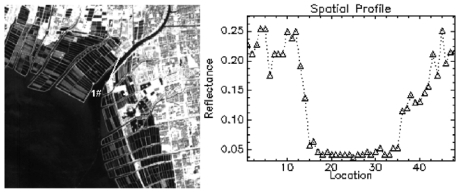
Variance range of water spatial reflectance profile at a Hyperion NIR band (864.35 nm).

The Hyperion imagery acquired was pre-processed according to the guides of Hyperion imagery pre-processing [17]. The first step was to fix the bad lines of imagery. There were a few Hyperion detectors found to be bad in over 60,000 detectors (256 pixels × 220 bands). Bad detectors were corrected by the use of neighbor interpolation. The second step was to retrieve water area in the imagery. River and coastal water (investigation object) reflectance was analyzed according to their spatial profile (Figure 2). Figure 2 represents the water spatial reflectance profile using a Hyperion NIR band (864.35nm). It demonstrates that their reflectance values are below 0.075 on Hyperion imagery before atmospheric correction. To help us to extract water regions in ArcGIS, area information was added, because the connectivity area of river is generally larger than other water bodies.

Finally, removal of atmospheric effects is especially important due to the low reflectance of water body. Hyperion imagery was performed using the Fast Line-of-sight Atmospheric Analysis of Spectral Hypercubes (FLAASH) software package in ENVI 4.0 (Research Systems Corp.). FLAASH is designed for atmospheric correction of hyperspectral and multispectral data [18]. It incorporates MODTRAN 4 radioactive transfer codes with all MODTRAN atmosphere and aerosol types to calculate a unique solution for each image. FLAASH also includes a correction for the adjacency effect, provides an option to compute a scene-average visibility (aerosol/haze amount) and utilizes advanced techniques for handling particularly challenging atmospheric conditions such as clouds. FLAASH produces results in terms of a scaled radiance reflectance that equals irradiance reflectance in the case of Lambertian surfaces. In this study, model parameters describing a mid-latitude winter atmosphere and rural aerosols together with automatic aerosol retrieval were used in FLAASH to correct the Hyperion imagery. Finally, geometric correction was performed by the nearest neighbor resample method based on a water system graph of 1:50,000 scale in the PRE (the water system graph is mapped in 2005) within 0.5 pixel departure.

**Figure 3.**
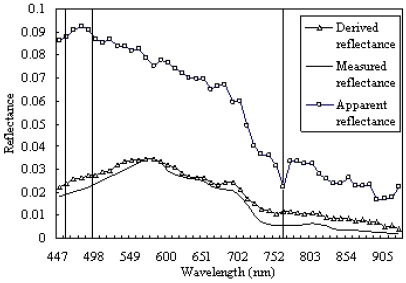
The comparison of the FLAASH-derived reflectance with the apparent reflectance, and with the measured Hyperion remote sensing reflectance of surface water in the PRE. The three vertical lines indicate blue, green and visible light bands from left to right in the Figure.

To validate the results of atmospheric correction of FLAASH, the comparison of the derived reflectance with FLAASH and apparent reflectance of EO-1 image was produced. From Figure 3, the apparent reflectance of surface water in PRE is higher in visible band (especially in blue band) than the measured reflectance, and decreases with wavelength increasing. However, the derived reflectance with FLAASH decreases in visible band, and the most distinct lowering occurs in blue band and green band. In the visible light band, the radiance distortion is mainly caused by Rayleigh scattering and diffused scattering in the atmospheric window. Rayleigh scattering decreases with band increasing with the strongest effect in blue band. The diffused scattering results from cloud particle and it has an effect on all wavelengths of visible light. So, the scattering radiance must be removed from image signal by atmospheric correction method, which results in the decrease of object reflectance in the visible band.

Additionally, the derived spectral curve with FLAASH is compared with in-situ spectral curve synchronous with EO-1 Hyperion satellite imagery acquisition. From Figure 3, the spectral values of surface water derived with FLAASH are a little higher than in-situ remote sensing reflectance, however, two spectral curves have same change trend with similar peak and crest (RMSE of 0.0039). First, the reflectance increases with band increasing, and arrives at peak near 580 nm. Then, decreases with band increasing, and arrives at another peak near 680 nm in the visible band. In conclusion, for surface water of PRE, the atmospheric correction based on FLAASH is effective by comparing the derived reflectance of EO-1 Hyperion imagery with in-situ reflectance.

## 3. Results

### 3.1. Relationship between In-situ Reflectance and Water Constituents

**Figure 4.**
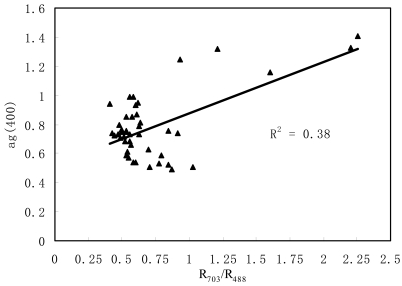
Correlation between ag(400) and reflectance ratio.

The study area covered a large variety of optically different waters. For example, the ag(400) sampled varied between 0.488 and 1.41 m^-1^. There were better results among the single band and band ratios and their varieties were obtained in reflectance ratios, especially for the correlation of reflectance with water constituents in the PRE. There was positive correlation between ag(400) and reflectance ratio R_703_/ R_488_(r = 0.62). However, the ag(400) of most sites varied between 0.5 and 1, so the linear relationship between ag(400) and the reflectance ratio need to be validated by more measurement data with high CDOM concentrations (Figure 4). Bowers *et al.* [19] showed from theory that there should be a linear relationship between ag(400) and the ratio of reflectance in the red and one other color, provided the effect of suspended particles on the optical signature is weak compared to that of CDOM. Thus, the relationship between ag(400) and the reflectance ratio was not rigorous, due to the strong influence of other water constituents (such as TSS) on ocean color in the PRE.

Based on the above analysis, the correlation of TSS concentration and reflectance was further examined. There were 44 TSS sample data synchronous with field spectra measurements on December 18, 19 and 21, 2006. TSS concentrations varied between 7.0 and 241.1, covering a large variety of optically different coastal waters.

**Figure 5.**
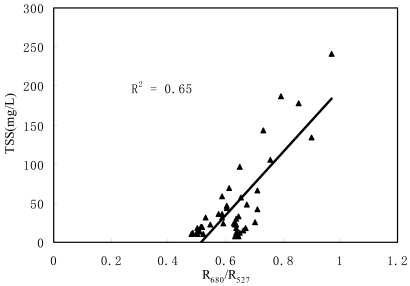
Correlation between TSS concentration and reflectance ratio.

Figure 5 shows a correlation of TSS against R_680_/R_527_ for all sites in the delta river ways and Lingding Bay. Remarkably, stations from a variety of locations and also over a range of TSS concentrations supported a good linear correlation between TSS and reflectance ratio of R_680_/R_527_. A least-squares linear regression on TSS against R_680_/R_527_ produced the following correlation model:

(4) $$\text{TSS}=403.49\times \frac{R_{680}}{R_{527}}-207.55({\text{R}}^{2}=0.65,\text{n}=44,\text{P}<0.001)$$

The 44 point data were used to develop the linear model, which could describe nearly 65% of the variability in the ratio R_680_/R_527_ with a range of TSS, 7.0–241.1 mg/L.

Equation four indicates that the reflectance ratio is positively proportional to the TSS concentration, which is in accordance with the other algorithms showing an increase in red band reflectance [20] and a decrease in blue-green band with increasing sediment concentration [21]. Moreover, according to previous research, the success of algorithms based on visible wavelength color ratios has been restricted to regions where mineral sediments are likely to dominate the optical signal, such as the Bay of Fundy [22], the Irish Sea [20] and the Menai Strait [23].

Up to now, the existing algorithms for determining water quality parameters in the open ocean waters are more robust and universal than those for coastal water. Coarsely spectral and spatial resolution imagery in spectral properties of river estuary waters, different optical characteristics of the water constituents in different estuary waters and the problem of imagery atmospheric correction, which result in present algorithms are not suitable for determining the water constituents in seawater intrusion reaches or estuary water. Thus, it is necessary to analyze the spectral properties of the water constituents based on in-situ hyper-spectral data and develop reliable algorithms for simultaneously retrieving the temporal and spatial distribution of various water parameters in the PRE.

### 3.2. Correlation between Salinity and CDOM

The correlation between ag (400) and salinity was analyzed from field data (Tables 1, 2 and 3). The ag (400) is significantly correlated with salinity in the studied field. Salinity decreases linearly with the increasing CDOM absorption coefficient. The correlation could be described by Equation (5), and involved data of 39 sample sites (including different marine intrusion river reaches and marine water) with a good linear correlation (R^2^ = 0.77).

However, five *in-situ* data points of lower salinity were discarded, which were from the Shenwan River way and were controlled by severe pollution of the Xihe waterway by industry and life.

**Figure 6.**
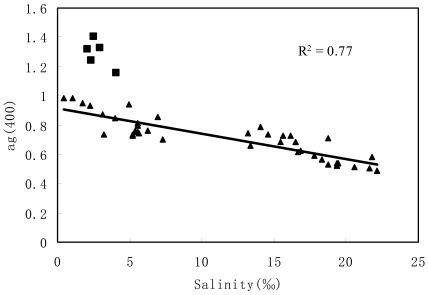
Correlation between salinity (‰) and CDOM absorption coefficients. Squares present five in-situ data, which were from the Shenwan River way and were controlled by severe pollution of the Xihe waterway by industry and life.

Usually, a linear correlation exists between the absorption coefficients of CDOM and salinity in estuary, indicating that freshwater was the primary source of CDOM [11]. So, CDOM was rooted upstream and in the adjacent area in the PRE. However, the linear correlation is not only regional but also time dependent. The slope of this line is determined by the concentration of CDOM at the point of fresh water origin (salinity = 0) [24]. This will undoubtedly vary both geographically and temporally with factors such as variations in the availability and type of organic matter, volume of and persistence of rainfall, size of watershed, etc. From Figure 6, compared to the results [10, 11], the slope of this regression line is closed but smaller than Hong's and this shows that the Pearl River discharge controls the slope of regression line between CDOM and salinity. Additionally, the relationship that was observed under a high salinity estuary does not work under lower salinity of marine intrusion river reach (salinity < 0.45‰) in the PRE [25].

(5) $$\text{Salinity}=-44.618\times g_{400}+43.5417\;({\text{R}}^{2}=0.77,\text{n}=39,\text{P}<0.001)$$

### 3.3. Correlation between Salinity and TSS

**Figure 7.**
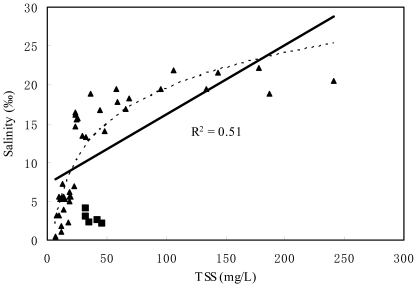
Correlation between salinity (‰) and total suspended solids (TSS) concentration (mg/L), and the black full line represent linear relationship between salinity and total suspended solids (TSS) concentration (R^2^ = 0.51).

At the same time, we examined the correlation between TSS and salinity based on *in-situ* TSS and salinity data (Tables 1, 2 and 3). The TSS concentration is significantly correlated with salinity in the PRE. Salinity increases with the increasing suspended sediment concentrations, while the slope of the curve is larger under the low suspended sediment concentrations and change of salinity is slow when suspended sediment concentrations arrive at a certain value (about 100 mg/L) (Figure 7).

The correlation could be described by Equation (6) and involved data of 39 sample sites with a better linear correlation (R^2^ = 0.51). However, five *in-situ* data points in Shenwan River way were discarded due to severe eutrophication. It suggested that the correlation between salinity and water constituents deserved further study under lower salinity conditions of marine intrusion river reach in the PRE. Moreover, a strong negatively exponential correlation between salinity and TSS (R^2^ = 0.84, P < 0.001) was built under low salinity (< 1.33‰) based on field data of December 18, 2005 [25].

Equation (6) indicates that concentrations of TSS are positively correlated with salinity, which is inconsistent with investigation results in Spring and Summer, 2003, in the Lingding Bay water of the PRE [26]. With the suspended particles, it has a different response to salinity during different water quality and anthropogenic activity conditions in estuary. As a whole, the correlation between TSS and salinity is limited by many conditions, such as tidal forcing, freshwater inflows, geometry of river network systems, bottom materials, etc. Each of these processes act on a different time scale, ranging from seconds to years, and affects suspended sediment concentration differently, depending on the local bathymetric and physical environment [27]. In the waterway of the PRE, tides result in salinity and suspended sediment increasing due to marine intrusion. However, freshwater containing a mass of dissolved and particulate materials arose mostly due to urban development, discharge into the South China Sea. The TSS concentrations of each river mouth can be clearly changed after the river flows through the delta plains where the urban land use extends quickly, especially during the dry season. Moreover, the suspended particulate properties of each river mouth also differ because the river receives different amounts of urban sewage [2]. Adding the large seasonal variation in runoff the Lingding estuarine hydrodynamics are commonly characterized as well stratified during the wet season and well mixed during the dry season with regard to the salinity field [28, 29]. The marine water wedge and turbidity maximum also shift seasonally [30, 31]. Estuarine circulation is mainly formed by the upper layer of brackish water that flows out and entrains the lower layer of high salinity water from the inner shelf which flows in at the bottom of the estuary. The net advection and tidal pumping mainly controlled the suspended sediment concentrations [32].

(6) $$\text{Salinity}=6.5669\times \ln\text{TSS}-10.727\;({\text{R}}^{2}=0.79,\text{n}=39,\text{P}<0.001)$$

During the field measurement on December 18, 19 and 21, in 2006 (during the dry season), a strong tidal action happened with a large tidal volume during the flood tide, which dominates the salinity of river. Therefore, it results in salinity increasing and suspended sediment increasing due to marine intrusion.

### 3.4. Mapping salinity and TSS concentrations from EO-1 Hyperion Imagery

**Figure 8.**
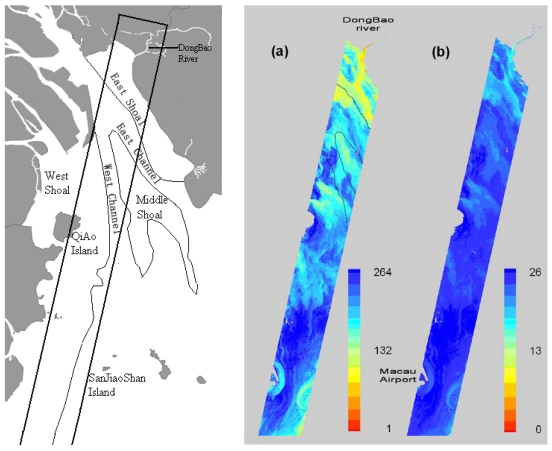
The left graph is sea graph of Lingding Bay; the right graph is products from the semi-empirical algorithms on December 21, 2006. (a) total suspended solid (mg/L). (b) salinity (‰).

According to the studies of Lee *et al.* [33] and Chen *et al.* [34], the ratio of irradiance reflectance is equal to the ratio of remote sensing reflectance. Thus these algorithms can be applied to retrieve TSS concentrations and salinity from the pre-processed Hyperion imagery. The ratio of the 33^rd^ band (681.2 nm) and the 18^th^ band (528.57 nm) of Hyperion imagery was selected for the retrieving of TSS concentrations and salinity using Equation 4 and Equation 6, and the retrieved TSS and salinity are shown in Figure 8.

Areas of Low concentrations of TSS are detected near the mouths of rivers, especially at the mouths of the HuMen waterway and Dongbao River [Figure 1 and Figure 8(a)]. The lowest retrieved value is under 5 mg/L. Other low values, near four mouths of PRE (from east to west, HuMen, JiaoMen, HongQiMen, HengMen) are below 80 mg/L. Low concentrations of TSS were also detected near island (Macau Airport and SanJiaoShan Island), with the value of 120 mg/L. The distribution of salinity was the same as that of TSS. Salinity of the mouths of the rivers is lower and salinity gradually increases further from the river mouth. For Dongbao River, that is the sole river in the Hyperion imagery, the surface salinity decreases from the mouth of the river to the upper river area. The lowest salinity is under 13‰, which is found in the Dongbao River.

Combining with the sea graph of Lingding Bay [Figure 8 (left)], the characteristic of distribution of TSS can be presented as follows: First, TSS concentrations in the west shoal are highest, the next is the middle shoal, and TSS concentration in the east shoal is lowest. Second, TSS concentrations of the west channel are higher than the east channel. However, TSS concentrations of east-west channels are higher than shoals, and the characteristic of distribution is same with other results, such as the quantitative estimation of multi-temporal remote sensing images during different seasons and tide period [35]. Salinity in the east shoal is lowest, the next is the middle shoal, and salinity in the west shoal is the highest. Moreover, salinity of east-west channels is higher than shoals, which was the same as that of TSS.

## 4. Discussions

Table 4 presents the inverse and measured TSS concentrations and salinity for validation. The concentration of TSS and salinity could be reasonably well estimated and the root mean square errors (RMSE) were small below 11 mg/L (relative error < 17%), although low concentration area was slightly overestimated and high concentration area was slightly underestimated. The reason is probably inhomogeneous atmospheric parameters. In this study, single values for the atmospheric parameters were used for the whole imagery. However, the atmospheric parameters should be different from place to place, in particular in the bay region near industrial cities. These retrieved results were similar to environmental bulletin of 2006 (TSS concentrations of river reaches are much lower near the mouth, and increase gradually further from the mouth) compiled by the Committee of Environmental Protection of Guangdong province in the PRE [36].

**Figure 9.**
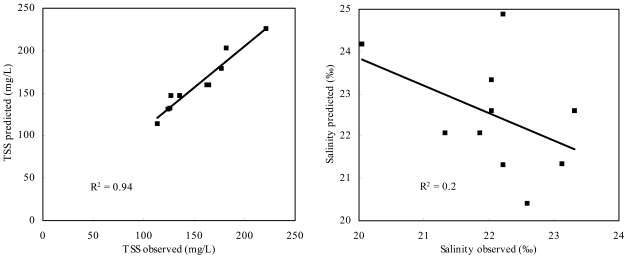
Correlations between the *in-situ* TSS concentrations and salinity data and estimated from an atmospherically corrected Hyperion image of PRE (Dec. 2006) using Equations (4) and (6). Ten stations data synchronous with EO-1 Hyperion imagery were used to validate TSS concentrations and salinity.

The comparison graphs of predicted and observed data (Figure 9) show the strong relationship (R^2^ = 0.94) between field and Hyperion estimated TSS concentrations (left graph), however, a weaker correlation between *in-situ* and Hyperion-estimated salinity (right graph) (R^2^ = 0.2). Several reasons are responsible for the weaker correlation. First, in-situ salinity varied rarely, between 20.05 and 23.31. Second, the synchronous filed measurement points were adjacent due to difficult acquisition condition (Figure 1), and this affects the reliability of validation results.

Hyperspectral data were very important in the selection of the PRE for field sampling and for construction of salinity model, because they allowed for a better characterization of the variations between the reflectance and water constitute parameters due to changes in water constituents. The new model of salinity has the potential to estimate the salinity variations of the complex network of river tributaries and channels as well as map marine water TSS concentrations and salinity anytime based on appropriate remote sensing imagery (such as EO-1 Hyperion imagery), which will largely improve the ability to inspect the phenomena of marine water intrusion in PRE. Furthermore, the new model could help us to better understand the mechanism of coastal marine water dynamics and to find an effective way to alleviate the influence of marine water intrusion into drinking water, and understand more about the situation of estuarine ecosystems.

## 5. Conclusions

In recent years, seawater intrusion into Pearl River occurs earlier and lasts longer, which creates a severe drinkable water security issue in the Peal River Estuary (PRE) in South China. However, there are no efficient methods that can quickly estimate the salinity variations in a large spatial scale such as in the PRE. Instead, such estimation has to rely on substantial field investigation in order to understand the spatial characteristics of the marine water intrusion into drinking water taking sites, but it is difficult to both access and afford with enough density distribution.

In this research, on the basis of the analysis of *in-situ* data from 44 sampling sites in the PRE, the spectral properties of estuarine water constituents (TSS and CDOM) were analyzed and the models between salinity and water constituents were built. According to the results, water constituents were feebly correlated with single waveband reflectance, but better correlated with reflectance ratio. TSS and CDOM concentrations increased with decreased reflectance in blue-green band due to the strong absorption by particulate and dissolved materials and increased with increased reflectance in red band. It affirms to optical properties of coastal water. Some significant correlations between in-situ reflectance ratios and TSS concentrations were observed based on synchronous in-situ spectra and marine water constituents' concentrations data on December 18, 19 and 21, 2006 in the PRE. In addition, we also observed two significant correlations (R^2^ > 77%, P < 0.001), one between TSS and surface salinity and the other between ag (400) and surface salinity. Combining with the salinity model built based on *in-situ* data on December 18, 2005, when salinity of river water is under the criterion (almost equal to intrusion seawater salinity value for drinkable freshwater, 250 mg/L) [25], we were able to develop a robust, universal and accurate method of inverting surface salinity from remotely sensed reflectance in the PRE. Thus, the new approach was applied for detecting TSS concentrations and surface salinity from EO-1 Hyperion imagery. Maps of TSS concentrations and salinity in the PRE were derived from Hyperion imagery based on the semi-empirical correlations in this study. The inversed result showed the high salinity in the channels and low salinity in the shoal in Lingding Bay. Additionally, the comparison between the synchronous *in-situ* observed data and the data retrieved from Hyperion imagery indicated that the empirical correlations are significant and powerful for mapping TSS concentrations and salinity in the PRE. However, the equations (4) and (6) are the regional empirical algorithms, which were built based on the data of river network area and Lingding Bay in the PRE. The models are applicable to monitor the TSS concentrations and salinity of river net area and Lingding Bay, namely for inspecting the saltwater intrusion reaches in the PRE.

## Figures and Tables

**Table 1.** The constituents of samples that were collected simultaneously with field spectrum measurement on December 18 2006 in the Humen waterway of PRE.

Location	H1	H2	H3	H4	H5	H6	H7	H8	H9	H10	H11	H12	H13	H14	H15	H16
ag(400)(/m)	0.71	0.73	0.66	0.68	0.68	0.73	0.73	0.78	0.74	0.86	0.82	0.95	0.87	0.93	0.99	0.99
TSS (mg/L)	36.6	23.2	29.4	24.0	24.6	23.2	25.4	48.6	32.4	22.4	18.2	11.4	7.6	17.6	12.0	7.0
Salinity(‰)	18.8	16.2	13.4	16.5	15.5	14.6	15.6	14.1	13.2	6.96	5.56	1.74	3.16	2.22	1.08	0.43

**Table 2.** The constituents of samples that were collected simultaneously with field spectrum measurement on December 19 2006 in the Modaomen waterway of PRE.

Location	M1	M2	M3	M4	M5	M6	M7	M8	M9	M10	M11	M12	M13	M14	M15	M16
		
ag(400)(/m)	1.32	1.24	0.74	0.85	0.72	0.79	0.76	0.71	0.77	0.74	0.76	0.74	1.16	0.94	1.33	1.41
TSS (mg/L)	46.5	35.6	10.1	13.8	10.9	10.2	19.8	12.7	19.1	14.2	11.8	14.8	32.1	18.4	32.7	42.1
Salinity(‰)	2.11	2.33	3.23	3.99	5.2	5.56	5.56	7.3	6.25	5.64	5.38	5.2	4.08	4.95	2.94	2.51

**Table 3.** The constituents of samples that were collected simultaneously with field spectrum measurement on December 21 2006 in the Lingding Bay of PRE.

Location	S1	S2	S3	S4	S5	S6	S7	S8	S9	S10	S11	S12
ag(400)(/m)	0.584	0.509	0.488	0.509	0.519	0.530	0.536	0.541	0.568	0.623	0.590	0.612
TSS (mg/L)	106	143.6	178.4	241.1	133.6	187	58	95.8	69.3	66.2	58.9	43.9
Salinity(‰)	18.49	21.59	22.12	20.57	19.38	18.78	19.38	19.44	18.30	16.81	17.77	16.69

**Table 4.** Comparison between observed and EO-1 Hyperion derived TSS concentrations and salinity.

**Site**	**E1**	**E2**	**E3**	**E4**	**E5**	**W1**	**W2**	**W3**	**W4**	**W5**	**RMSE**
TSS observed	162.4	123.8	126.4	113.3	164	220.7	127.4	135.6	177.2	182.2	10.8
TSS predicted	159.99	131.7	132.2	114.7	160	226.3	147.8	147.8	178.7	203.4	
Salinity observed	22.04	22.22	23.13	22.58	23.31	22.22	21.32	21.86	22.04	20.05	1.89
Salinity predicted	22.60	21.32	21.35	20.42	22.60	24.88	22.08	22.08	23.33	24.18	

## References

[1] Preisendorfer R.W., Mobley C.D. (1984). Direct and inverse irradiance models in hydrologic optics. Limnol. Oceanogr..

[2] Wang Z.G., Liu W.Q., Li H.B. (2006). Analysis of CDOM spatial distribution variations in Chaohu Lake and its soures by three dimensional fluorescence excitation-emission matrix. Acta Sci. Cirum..

[3] Xia X., Li Y., Yang H., Wu C., Sing T., Pong H. (2004). Observations on the size and settling velocity distributions of suspended sediment in the Pearl River Estuary, China. Cont. Shelf Res..

[4] Weidemann A.D., Johnson D.J., Holyer R.J., Pegau W.S., Jugan L.A., Sandidge J.C. (2001). Remote imaging of internal solitons in the coastal ocean. Remote Sens. Environ..

[5] Feng H., Campbell J.W., Dowell M.D., Moore T.S. (2005). Modeling spectral reflectance of optically complex waters using bio-optical measurements from Tokyo Bay. Remote Sens. Environ..

[6] Brando V.E, Dekker A.G. (2003). Satellite Hyperspectral remote sensing for estimating estuarine and coastal water quality. IEEE Trans. Geosci. Remote Sens..

[7] Miller R.L., McKee B.A. (2004). Using MODIS Terra 250 m imagery to map concentrations of total suspended matter in coastal waters. Remote. Sens. Environ..

[8] Hu C., Chen Z., Tonya D., Clayton P., Swarzenski J.C., Brock F.E., Muller K. (2004). Assessment of estuarine water-quality indicators using MODIS medium-resolution bands: Initial results from Tampa Bay, FL. Remote Sens. Environ..

[9] Yang J., Chen C. (2007). An optimal algorithm for retrieval of chlorophyll, suspended sediments and gelbstoff of case II waters in Zhujiang River estuary. J. Trop. Oceanogr..

[10] Chen Z., Li Y., Pan J.M. (2004). Distributions of the optical properties of colored dissolved organic matter and dissolved organic carbon in the Pearl River Estuary. Cont. Shelf Res..

[11] Hong H., Wu J., Shang S., Hu C. (2005). Absorption and fluorescence of chromophoric dissolved organic matter in the Pearl River Estuary, South China. Mar. Chem..

[12] Chen C., Pan Z., Shi P. (2003). Simulation of sea water reflectance and its Application in Retrieval of Yellow Substance by Remote Sensing Data. J. Trop. Oceanogr..

[13] http://gz.dayoo.com/gb/content/2006-01/09/content_2370057.htm. Accessed 9 January 2006

[14] Ministry of Environmental Protection of the People's Republic of China (2003). Technical specifications requirements for monitoring of surface water and waste water, “HJ/T 91-2002”. Chinese Environ sci. publication..

[15] Mitchell B.G., Bricaud A., Carder K., Cleveland J., Ferrari G.M., Gould R., Kahru M., Kishino M., Maske H., Moisan T., Moore L., Nelson N., Phinney D., Reynolds R.A., Sosik H., Stramski D., Tassan S., Trees C., Weidemann A., Wieland J.D., Vodacek A., Fargion G.S., Mueller J.L., McClain C.R. (2000). Determination of spectral absorption coefficients of particles, dissolved material and phytoplankton for discrete water samples. Ocean Optics Protocols For Satellite Ocean Color Sensor Validation.

[16] Tang J., Tian G. (2004). The Methods of Water Spectra Measurement and Analysis: Surface-Water Method. J. Remote Sens..

[17] http://eo1.usgs.gov/. Accessed 25 June 2008

[18] Adler-Golden S.M., Matthew M.W., Bernstein L.S., Levine R.Y., Berk A., Richtmeier S.C. Atmospheric correction for short-wave imagery based on MODTRAN 4.

[19] Bowers D.G., Harker G.E.L., Smith P.S.D., Tett P. (2000). Optical properties of a region of freshwater influence (the Clyde Sea). Estuar. Coast. Shelf Sci..

[20] Binding C.E., Bowers D.G., Mitchelson-Jacob E.G. (2003). An algorithm for the retrieval of suspended sediment concentrations in the Irish Sea from SeaWiFS ocean colour satellite imagery. Int. J. Remote Sens..

[21] Gong C., Yin Q., Kuang D., Tian H. (2006). Study on the spectral reflectivity models of deffirent water quality parameters in Huangpu River. J. Infrared Millim. Waves.

[22] Topliss B.J. (1986). Spectral variations in upwelling radiant intensity in turbid coastal waters. Estuar. Coast Shelf S..

[23] Kratzer S., Bowers D., Tett P.T. (2000). Seasonal changes in colour ratios and optically active contituents in the optical Case-2 waters of the Menai Strait, North Wales. Int. J. Remote Sens..

[24] Binding C.E., Bowers D.G. (2003). Measuring the salinity of the Clyde Sea from remotely sensed ocean colour. Estuar. Coast Shelf S..

[25] Fang L.G., Chen S.S., Zeng Y.H., Zhang L.X., Chen X.H., Chen Y.D., Xia J., Zhang H. (2008). Monitoring marine water intrusion into river by ALI image - A case study in Pearl River estuary. Hydrological Sciences for Managing Water Resources in the Asian Developing World.

[26] Yang M., Lin Q., Lv X., Cai W. (2005). Distribution character istics of suspended substance in the Lingdingyang water of the Pearl River Estuary. South China Fish. Sci..

[27] Cloern J.E., Nichols F.H. (1985). Time scales and mechanisms of estuarine variability, a synthesis from studies of San Francisco Bay. Temporal Dynamics of an Estuary-San Francisco Bay.

[28] Ying Z.F., Chen S.G. (1983). Mixture feature of freshwater and seawater in Lingding Estuary of Pearl River. Acta Oceanologica Sinica.

[29] Dong L., Su J., Wong L.A., Cao Z., Chen J.C. (2004). Seasonal variation and dynamics of the Pearl River plume. Cont Shelf Res.

[30] Tian X.P. (1986). A study on turbidity maximum in Lingdingyang Estuary of the Pearl River. Trop. Oceanogr..

[31] Deng M., Huang W., Li Y. (2002). Data collection of remote sensing derived suspended sediment concentration in Zhujiang River estuary. Oceanol. Etlimnol. Sin..

[32] Chen Z.S. (1993). Analysis on longitudinal net circulations and material fluxes in Lingding estuary, Pearl River and adjacent inner shelf waters. Trop. Oceanogr..

[33] Lee Z., Carder K.L., Hawes S.K., Steward R.G., Peacock T.G., D. C.O. (1994). Model for the interpretation of hyperspectral remote sensing reflectance. Appl. Opt..

[34] Chen C., Shi P. (2002). Estimation of chlorophyll-a concentration in the Zhujiang Estuary from seaWiFS data. Acta Oceanol. Sin..

[35] Li X. (1992). A United equation for remote sensing quantitative analysis of suspended sediment and its application at Zhujiang River Estuary. Remote Sens. Environ. China.

[36] http://www.gdepb.gov.cn/. Accessed 1 January 2007

